# Predictive performance of parent-metabolite population pharmacokinetic models of (S)-ketamine in healthy volunteers

**DOI:** 10.1007/s00228-021-03104-1

**Published:** 2021-02-11

**Authors:** M. E. Otto, K. R. Bergmann, G. Jacobs, Michiel J. van Esdonk

**Affiliations:** 1 Centre for Human Drug Research, Leiden, The Netherlands; 2 Leiden Academic Centre for Drug Research, Leiden University, Leiden, The Netherlands; 3 Department of Psychiatry, Leiden University Medical Centre, Leiden, The Netherlands

**Keywords:** (S)-ketamine, (S)-norketamine, Population pharmacokinetics, Pharmacokinetic model, External validation

## Abstract

**Purpose:**

The recent repurposing of ketamine as treatment for pain and depression has increased the need for accurate population pharmacokinetic (PK) models to inform the design of new clinical trials. Therefore, the objectives of this study were to externally validate available PK models on (S)-(nor)ketamine concentrations with in-house data and to improve the best performing model when necessary.

**Methods:**

Based on predefined criteria, five models were selected from literature. Data of two previously performed clinical trials on (S)-ketamine administration in healthy volunteers were available for validation. The predictive performances of the selected models were compared through visual predictive checks (VPCs) and calculation of the (root) mean (square) prediction errors (ME and RMSE). The available data was used to adapt the best performing model through alterations to the model structure and re-estimation of inter-individual variability (IIV).

**Results:**

The model developed by Fanta et al. (*Eur J Clin Pharmacol* 71:441–447, 2015) performed best at predicting the (S)-ketamine concentration over time, but failed to capture the (S)-norketamine *C*_max_ correctly. Other models with similar population demographics and study designs had estimated relatively small distribution volumes of (S)-ketamine and thus overpredicted concentrations after start of infusion, most likely due to the influence of circulatory dynamics and sampling methodology. Model predictions were improved through a reduction in complexity of the (S)-(nor)ketamine model and re-estimation of IIV.

**Conclusion:**

The modified model resulted in accurate predictions of both (S)-ketamine and (S)-norketamine and thereby provides a solid foundation for future simulation studies of (S)-(nor)ketamine PK in healthy volunteers after (S)-ketamine infusion.

**Supplementary Information:**

The online version contains supplementary material available at 10.1007/s00228-021-03104-1.

## Introduction

Although ketamine has been used in the clinic as anesthetic for half a century, interest in this compound has reignited and been increasing because of its possible application as new drug modality for pain and persistent depression at low doses [1–5]. This has already resulted in the authorization of intranasally administered (S)-ketamine as treatment for patients with treatment-resistant depression by the European Medicines Agency (EMA) in 2019 [6, 7].

Ketamine is an arylcyclohexylamine and has two enantiomers, (R)-ketamine and (S)-ketamine. It is mainly metabolized into norketamine, though other metabolites have been reported as well [8, 9]. (S)-ketamine is transformed into the norketamine metabolite 20% faster compared to (R)-ketamine [10, 11]. Both enantiomers of ketamine and norketamine induce anesthetic and analgesic effects through inhibition of the N-methyl-D-aspartate (NMDA) receptor, a key player in neurotransmitter signaling. The highest potency for this receptor is shown by (S)-ketamine, which is why most research so far focused on this enantiomer. Its affinity for the NMDA receptor is approximately 5 times higher compared to (R)-ketamine and even approximately 8 times higher when comparing the norketamine enantiomers [9, 12]. However, discussion remains on whether the demonstrated effect of ketamine on depression is also exclusively mediated through this receptor [9, 13].

Since ketamine, and (S)-ketamine in particular, has been widely used in the clinic, information on its pharmacokinetic (PK) properties is abundant [14]. This information is valuable for the design of future studies investigating the pharmacokinetic-pharmacodynamic (PKPD) relationship of (nor)ketamine’s antidepressant effects. Especially when the PKPD data have been used to develop non-linear mixed effects (population) models, simulations of new clinical trial scenarios can be performed to explore correct dosing and sampling regimens before the start of the actual trial. Several population PK models of ketamine have already been reported and used for this purpose [15, 16].

The available population PK models on ketamine are diverse in terms of administered and measured compound, being either racemic, (S)-ketamine or (R)-ketamine, but also in whether the PK of metabolites such as norketamine is described as well [17–33]. An important limitation in the cross-application of these PK models is the assumption that enantiomer-specific PK remains similar when administered as racemate or separate compounds. Evidence undermining this assumption was presented by Ihmsen et al. [34]*.* Their study suggested that the presence of (R)-ketamine inhibits (S)-ketamine clearance after racemic administration because of competition for metabolism. However, when Kamp et al. tested the infusion of racemic or (S)-ketamine as a covariate during the development of their population PK model, no significant differences were found [24].

In a second study, Kamp et al. approached the issue of model diversity with the development of a meta-analysis PK model based on 18 previously reported models and a population analysis PK model based on 14 raw datasets, thereby combining all the information that is already available [25]. Even though they reported the weighted mean distribution volume and clearance of norketamine, the formation rate and pharmacokinetics of the metabolite were not further described in both the meta-analytical and population analysis PK models. Interest in the compound and the secondary metabolites hydroxynorketamine and dehydroxynorketamine is increasing, as they have shown to also be pharmacologically active [9, 35]. This is especially important to consider when investigating oral administration of ketamine, as norketamine concentrations will be higher due to the first-pass effect and highlights the need for correct model predictions of its PK profile [27].

To design a study using a model-based approach, the ability of the selected model to predict data without bias and within a reasonable range of variability needs to be trustworthy. For this purpose, the predictive performance of a model can be validated with an external dataset. External validation is an important step in the cycle of model development and optimization, but is not often performed due to lack of an external dataset [36, 37]. Therefore, the objective of this study was to assess and compare the predictive performances of available population PK models of (S)-(nor)ketamine with two in-house datasets collected in healthy volunteers. A secondary goal was to check whether, and which, improvements were required for the best performing model for an adequate description of the concentrations over time.

## Methods

### Model selection and comparison

A literature search was performed for population models describing the PK of (S)-ketamine and (S)-norketamine concentrations. In order to be selected for validation, models had to describe the PK (1) in adults, (2) of both (S)-ketamine and (S)-norketamine, and (3) after administration of (S)-ketamine (i.e., not racemic or (R)-ketamine). The selected models were implemented in NONMEM based on the information presented in the publications and sanity checks (e.g., simulation of similar doses, reproduction of reported figures) were performed to verify correct implementation. Clearance (CL) and central volume of distribution (*V*_d_) parameter estimates and corresponding variability were compared between models by plotting their simulated distributions.

### In-house datasets

In-house data of two clinical trials (further mentioned by their study numbers CHDR1311 and CHDR1016) performed at the Centre for Human Drug Research (CHDR, Leiden, The Netherlands) were available. Study details, data, and analysis have previously been published elsewhere and a short description of the study design will be provided here (Table 1) [15, 38]. Both studies were approved by the Medical Ethics Committee of the Leiden University Medical Centre (LUMC, Leiden, The Netherlands) and adhered to the Declaration of Helsinki and were executed following Good Clinical Practice (ICH-GCP) guidelines. Written informed consent of the volunteers was obtained prior to inclusion in the study.

**Table 1:** Summary of the study designs, population demographics (mean ± SD), and analysis methods of in-house data available from studies CHDR1311 and CHDR1016 on (S)-(nor)ketamine

	CHDR1311	CHDR1016
Study design		
Administered drug	(S)-ketamine	(S)-ketamine
Infusion dose and duration	10 mg in 30 min	Pre-amendment (*N* = 4)* *t* = 0: 0.04 mg/kg (bolus) *t* = 0-6 min: 0.7 mg/kg/h (females +5%) *t* = 7-29 min: 0.45 mg/kg/h (females +10%) *t* = 30-120 min: 0.3 mg/kg/h (females +15%) Post-amendment (*N* = 27)* *t* = 0: 0.026 mg/kg (bolus) *t* = 0-14 min: 0.425 mg/kg/h (females +5%) *t* = 15-39 min: 0.275 mg/kg/h (females +10%) *t* = 40-120 min: 0.15 mg/kg/h (females +15%)
PK-sampling schedule (h)	0.5 - 1 - 2 - 3 - 4 - 5 - 6 - 7 - 8 - 9 - 10	0.5 - 1 - 1.5 - 2 -2.5 - 4.5 - 5.0 - 5.5
Sampling methodology	Venous	Venous
Analysis method	LC–MS/MS	HPLC-UV
LLOQ (ng/mL)	(S)-ketamine: 1.00 ng/mL (S)-norketamine: 0.50 ng/mL	10 ng/mL
Samples (n) (BLQ)	268 (0%)	864 (5.9%)
Demographics		
Population	Healthy volunteers	Healthy volunteers
N (male)	17 (9)	31 (17)
Age (year)	23.0 ± 3.6	23.6 ± 5.1
BMI	21.6 ± 2.0	22.4 ± 2.0
Weight (kg)	68.0 ± 7.2	71.3 ± 8.5
Registration		
Clinical trial number (EudraCT)	2013-003443-28	2010-022203-21
Approval by Ethics Committee (CCMO)	NL46000.058.13	NL33486.058.10

*LC-MS/MS* liquid chromatography–mass spectrometry, *HPLC-UV* high-performance liquid chromatography with ultraviolet detection, *LLOQ* lower limit of quantification, *BLQ* below limit of quantification, *CCMO* Central Committee on Research Involving Human Subjects

*CHDR1016 consisted of two occasions, the high (S)-ketamine concentration infusion was double the amount of the low (S)-ketamine concentration infusion, which is shown here

In both studies, healthy volunteers received an intravenous infusion of (S)-ketamine. In CHDR1311, 10 mg of (S)-ketamine was administered to 17 healthy volunteers as a 30-min infusion. Study CHDR1016 consisted of two occasions, in which either a low or high (S)-ketamine concentration infusion was administered at different infusion rates for 2 h in 31 healthy volunteers. The dosing schedule of CHDR1016 was adapted during the study due to adverse effects. Blood samples were collected up to 5.5 and 10 h after the end of infusion in CHDR1016 and CHDR1311, respectively.

### External validation

The predictive performances of the models were assessed with confidence interval visual predictive checks (ciVPC) and by calculation of the mean prediction error (ME) and root mean square prediction error (RMSE), based on the data of CHDR1311 and individual predictions. The best performing model was also evaluated with a prediction corrected visual predictive check (pcVPC) based on the data of CHDR1016 as another external validation step on a different dataset. VPCs were created for each model by simulating the corresponding study design 1000 times. The median concentration, corresponding 80% prediction intervals, ME, and RMSE were calculated for each simulation. The calculated measures of all simulations were then combined to determine their 95% confidence interval (CI) [39]. The data presented in the pcVPC was transformed to account for differences in dosing regimens, which allows for interpretation of the predictions over time [40]. A model was selected as best performing if ME and RMSE values were relatively low compared to other models and if the ciVPC showed the best agreement with the data in the typical trend over time and its ability to capture the existing variability in the data, based on overlap between the observed median and prediction intervals and their simulated 95% CI.

### Model redevelopment

If required due to structural misspecifications identified by the VPCs, the selected model for (S)-ketamine and (S)-norketamine PK was to be further refined by structural modifications and re-estimation of inter-individual variability (IIV) based on data of CHDR1311 and CHDR1016. Samples below the lower limit of quantification were removed from model development. Multiple structural models, with or without transit compartments for metabolite formation, were explored following a sequential modeling approach. Due to parameter identifiability issues, it was assumed that (S)-ketamine was fully metabolized into (S)-norketamine. Inclusion of IIV (eta~*N*(0,*ω*^2^)) was done following a forward inclusion procedure on all parameters, after which between-occasion variability (BOV) and covariance structures (omega blocks) were also tested if applicable. An exploratory covariate analysis of age, BMI, weight, and sex was performed by visual exploration of empirical Bayesian estimates (EBEs) of eta versus each covariate and numerically by calculation of the Pearson’s correlation coefficient. In case an allometric relationship was included in the base model, the parameter in question was to be scaled to a typical weight of 70 kg. Inclusion of a covariate in the model was considered when it was biologically plausible, in line with the selected base model, and did not worsen model performance. Additive, proportional, and combined error structures were tested to describe the residual unexplained variability (*ε*~*N*(0,*σ*^2^)). Inclusion of individual parameters or structural components had to be supported by well-distributed goodness-of-fit (GOF) plots, a significant drop in objective function value (dOFV ≤ −6.64, *p* = 0.01), low relative standard errors (RSE <50%), the condition number (<1000), and eta shrinkage (<30%), and was to be evaluated with a pcVPC.

### Software

Data transformations and visualization were performed in R (V3.6.1) [41]. Population modeling was performed using NONMEM (version 7.3, ICON Development Solutions, Hanover, MD) [42] in conjunction with PsN [43].

## Results

### Model selection and comparison

**Fig. 1:**
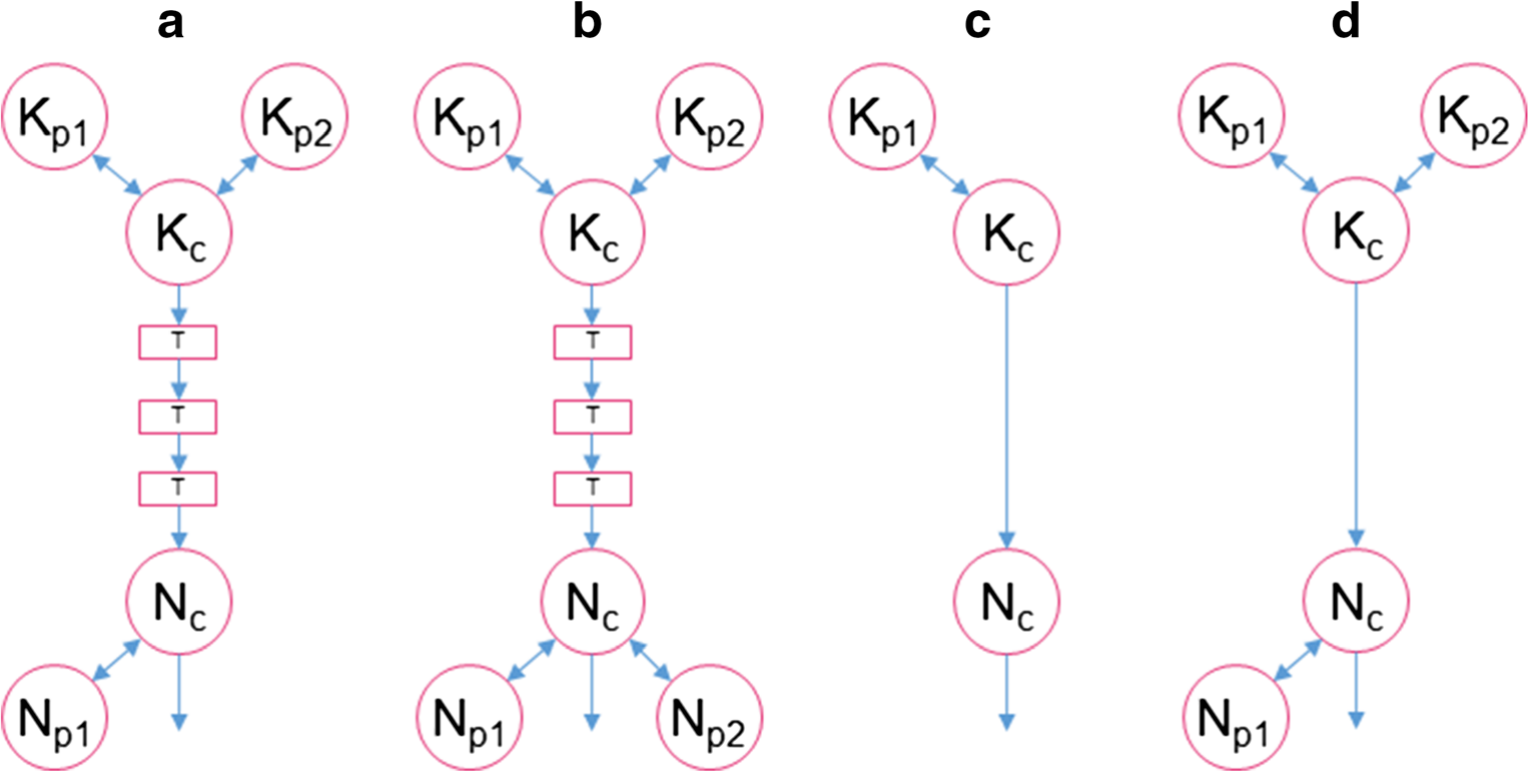
Schematic representation of (S)-(nor)ketamine literature PK model structures and the final redeveloped model structure. *K*_c_ and *N*_c_ depict the central (S)-ketamine and (S)-norketamine compartments; *K*_p1_, *K*_p2_, *N*_p1_, and *N*_p2_ are the first and second (S)-(nor)ketamine peripheral compartments; *T* represents the number of transit compartments. **a** Sigtermans et al., Noppers et al., and Jonkman et al. **b** Fanta et al. **c** Dahan et al. **d** Modified model structure of Fanta et al. based on CHDR1311 and CHDR1016 [18, 26, 27, 30, 31]

Five models were obtained from literature which met the predefined criteria [18, 26, 27, 30, 31]. Information on study designs, subject demographics, and applied dosing regimens is provided in Table 2. A schematic representation of the model structures is presented in Fig. 1. Model structures ranged in complexity, with two to three compartments for (S)-ketamine distribution and one to three compartments for (S)-norketamine distribution, while also including up to three transit compartments to describe the metabolism from ketamine to norketamine. Model development by Noppers et al. and Jonkman et al. was based on the previous published structure of Sigtermans et al. All models described PK in healthy volunteers, except for Dahan et al. [23], which studied ketamine administration in patients with complex regional pain syndrome type 1. All models were developed on data of intravenously administered (S)-ketamine, on top of which Jonkman et al. used data of inhaled (S)-ketamine and Fanta et al. studied oral administration.

**Table 2:** Summary of the study designs and population demographics of selected (S)-(nor)ketamine PK models from literature. *CRPS-1* complex regional pain syndrome type 1

	Sigtermans et al. [18]	Dahan et al. [30]	Noppers et al. [31]	Fanta et al. [27]	Jonkman et al. [26]
Study design					
Administered drug	(S)-ketamine	(S)-ketamine	(S)-ketamine	(S)-ketamine	(S)-ketamine
Mode of administration	Intravenous infusion (2h)	Intravenous infusion (100h)	Intravenous infusion (2h)	Intravenous bolus or oral dose	Inhalations followed by intravenous infusion (0.33 h)
Dose	0.95 mg/kg	0.07 mg/kg/h to 0.29 mg/kg/h	0.57 mg/kg and 1.14 mg/kg	Bolus: 0.125 mg/kg Oral: 0.25 mg/kg	Inhalation: 25 - 37.5 - 50.0 mg or 25 - 50 - 100 mg Infusion: 20 mg
PK-sampling schedule (h)	0.25 - 0.5 - 0.75 - 1 - 1.25 - 1.5 - 1.75 - 2 - 2.03 - 2.08 - 2.17 - 2.25 - 2.5 - 2.75 - 3 - 3.25 - 3.5 - 4 - 4.5 - 5	4 times a day, at change of infusion or at random	0 - 0.08 - 0.17 - 0.25 - 0.5 - 0.75 - 1 - 1.25 - 1. 5 - 1.75 - 2 - 2.08 - 2.16 - 2.25 - 2.5 - 3 - 3.5 - 4 - 4.5 - 5	0.04 - 0.08 - 0.17 - 0.25 - 0.33 - 0.5 - 0.75 - 1 - 1.5 - 2 - 4 - 6 - 8 - 12 - 23.5	0.03 - 0.07 - 0.13 - 0.17 - 0.25 - 0.33 - 0.37 - 0.4 - 0.5 - 0.67 - 1 -1.33 - 2 - 3
Sampling methodology	Arterial	Venous	Arterial	Venous	Arterial
Demographics					
Population	Healthy volunteers	CRPS-1 patients	Healthy volunteers	Healthy volunteers	Healthy volunteers
N (male)	20 (10)	30 (8)	20 (20)	11 (11)	19 (10)
Age (year)	21.7 ± 0.5	46 ± 12	22.0 (20-29)	22.3 ± 2.4	24 ± 2
BMI (kg/m^2^)	22.1 ± 0.5	n/a	22.2 (19-28)	23.6 ± 1.8	22 ± 2
Weight (kg)	69.4 ± 2.4	79 ± 19	Not reported	77 ± 7.5	Not reported

**Fig. 2:**
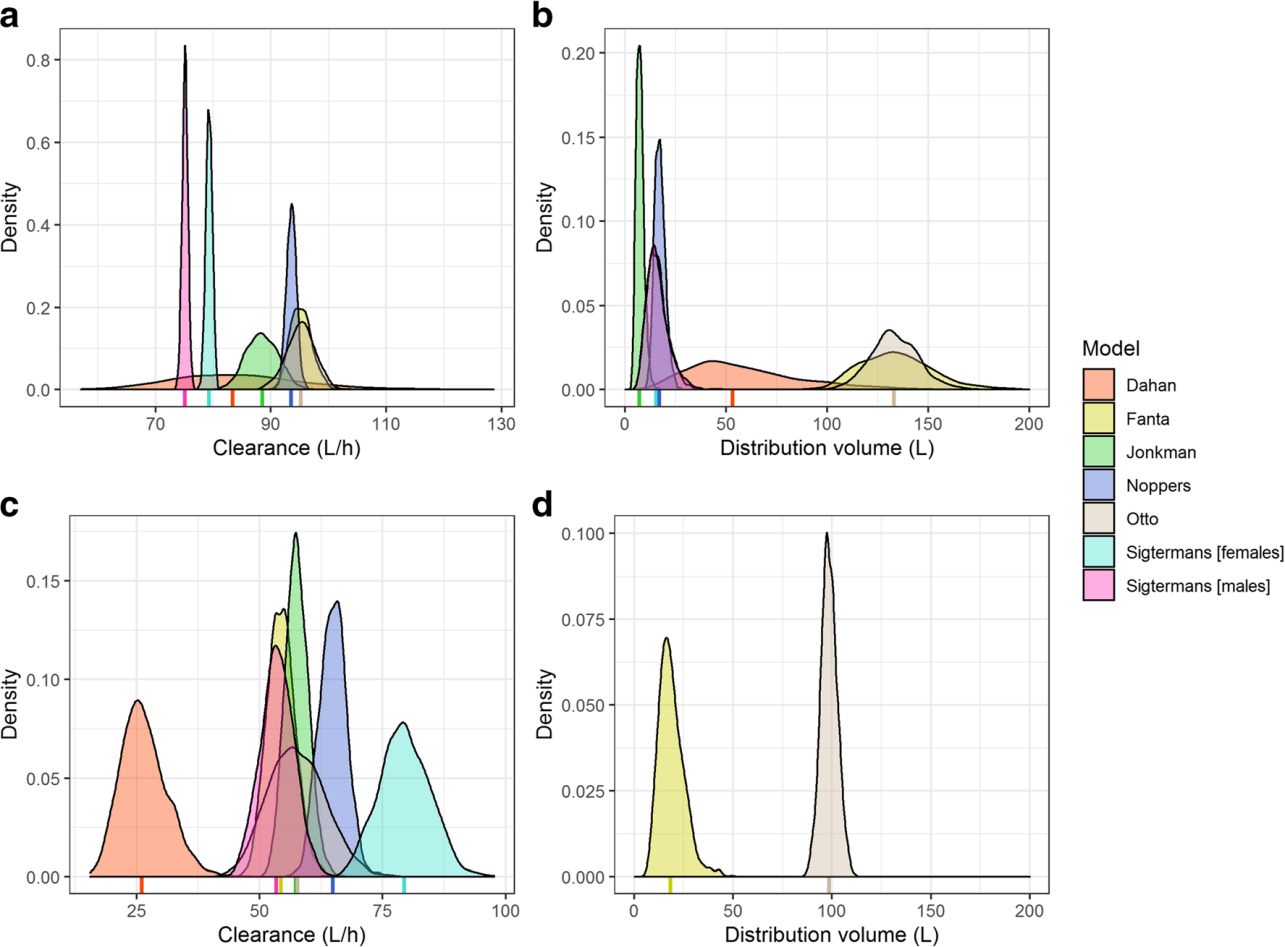
Simulated distributions of the individual clearance and central distribution volume parameters of selected literature PK models and the final redeveloped model describing (S)-ketamine (**a** and **b**) and (S)-norketamine (**c** and **d**) [18, 26, 27, 30, 31]. Individual parameter values were simulated 1000 times as Pi = TVP*exp(eta_IIV_ + eta_BOV_), in which eta represents the IIV or BOV when reported and is normally distributed with mean 0 and variance *ω*^2^, and TVP represents the parameter value of a typical individual, being indicated with a vertical line below the corresponding distribution. Sigtermans et al. included sex as covariate on CL but not on *V*_d_, which is why the (S)-ketamine *V*_d_ distributions of Sigtermans (males) and Sigtermans (females) overlap. The population parameters of the final redeveloped model describing (S)-ketamine were fixed to the values reported by Fanta et al.

The estimated model parameters were simulated with their corresponding typical value, IIV, and BOV (when applicable) which are shown in Fig. 2. It can be observed that there is a high level of variability of CL and *V*_d_ in the model of Dahan et al., which can be explained by their rather flexible study design and variable population demographics. The largest difference in parameter distributions occurs between (S)-ketamine *V*_d_ distributions, as the model of Fanta et al. has much higher values and variability compared to others (Fig. 2b). For (S)-ketamine CL, the distribution of Fanta et al. does overlap with other models, whereas those of Sigtermans et al. do not (Fig. 2a), but the relative difference is much less as with *V*_d_. A similar conclusion holds for the simulated CL of (S)-norketamine by Dahan et al. (Fig. 2c). Lastly, all models assumed (S)-norketamine *V*_d_ to be equal to (S)-ketamine *V*_d_, except for Fanta et al. who has estimated the parameter (Fig. 2d). Still, the estimated distribution of (S)-norketamine *V*_d_ is in a similar range as the (S)-ketamine *V*_d_ distributions of the other models (Fig. 2b versus Fig. 2d).

### External validation

**Fig. 3:**
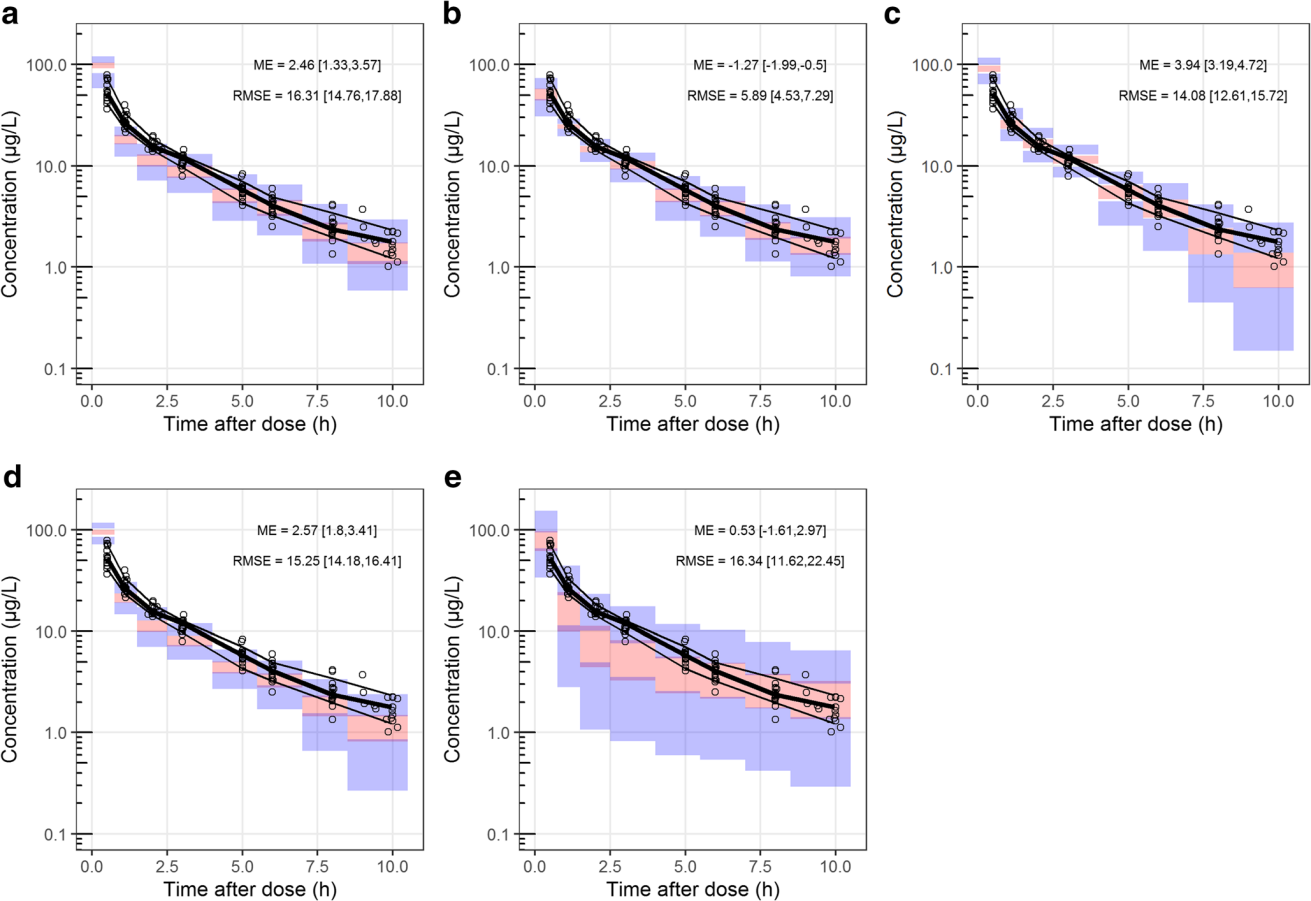
Confidence interval visual predictive checks (ciVPC) of (S)-ketamine model predictions of CHDR1311 data. ME = mean prediction error, RMSE = root mean squared prediction error (reported as their mean values with 95% CI in μg/L). **a** Jonkman et al. **b** Fanta et al. **c** Sigtermans et al. **d** Noppers et al. **e** Dahan et al. [18, 26, 27, 30, 31]. The thick and thin black lines represent the median and 80% intervals of observed data. The pink and purple rectangles represent the 95% confidence intervals around the median and 80% prediction intervals of the predicted data. The open dots show the observed concentrations

**Fig. 4:**
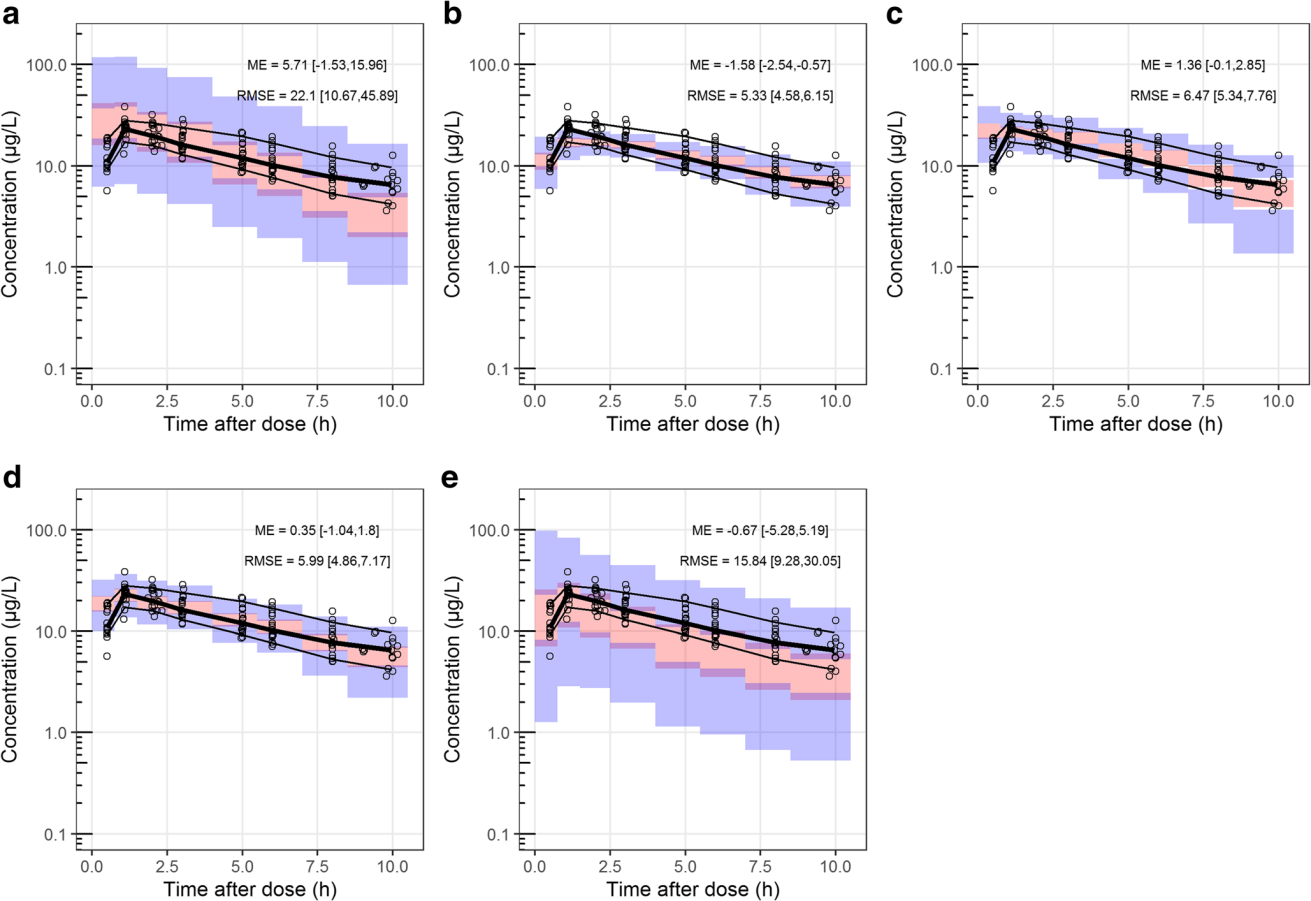
Confidence interval visual predictive checks (ciVPC) of (S)-norketamine model predictions of CHDR1311 data. ME = mean prediction error, RMSE = root mean squared prediction error (reported as their mean values with 95% CI in μg/L). **a** Jonkman et al. **b** Fanta et al. **c** Sigtermans et al. **d** Noppers et al. **e** Dahan et al. [18, 26, 27, 30, 31]. The thick and thin black lines represent the median and 80% intervals of observed data. The pink and purple rectangles represent the 95% confidence intervals around the median and 80% prediction intervals of the predicted data. The open dots show the observed concentrations

The data of CHDR1311 was predicted with the five selected models of which the ciVPCs are shown in Figs. 3 and 4. The three models with equivalent structures (Sigtermans et al. (2009), Jonkman et al. (2017) and Noppers et al. (2011)) have a similar predictive performance for (S)-ketamine, as seen by the general trend in the median-predicted concentrations in Fig. 3a, c, and d respectively. (S)-ketamine simulations with these models resulted in high ME and RMSE, which can be attributed to their overprediction of the *C*_max_. Furthermore, the median of the observed concentrations moves outside the model-predicted 95% CI over time, indicating overestimations of the elimination or metabolism of (S)-ketamine. Due to the lower concentrations at this timepoint, the ME and RMSE values are less affected by this discrepancy. These models overpredict the first observations of (S)-norketamine at 0.5h as well (Fig. 4a, c, and d). In this case however, the upper boundary of the 95% CI of the ME is much higher for Jonkman et al. compared to the other two models. This is likely to be caused by the high variability of the model, which is also indicated by the RMSE.

The overprediction of (S)-ketamine’s *C*_max_ is also present in the ciVPC of the model predictions by Dahan et al. (Fig. 3e). In contrast to the models discussed before, the ME is still relatively low because the overprediction after the start of administration is offsetted by the underprediction from 1-5 h after infusion. This latter disagreement in predicted and observed median concentrations suggests overestimated distribution to peripheral tissues rather than elimination. Furthermore, the predicted variability in (S)-ketamine is estimated too high by Dahan et al., which resonates in the large 95% CI of the RMSE.

The model presented by Fanta et al. performed best at predicting (S)-ketamine concentrations, which can be concluded visually by the agreement in simulated and observed data and quantitatively through the low ME and RMSE (Fig. 3b). (S)-norketamine simulations lead to the same conclusion, though the underestimation of the *C*_max_ resulted in a lower ME compared to Noppers et al. (Fig. 4b and d). These results show that the model by Fanta et al. was most suitable for further exploration with the second in-house dataset of study CHDR1016 (Figure S1). Similar to the first dataset, the model was able to predict the observations accurately, with the only irregularities being an overprediction of (S)-ketamine concentrations approximately 2h before infusion stopped and a similar underprediction of (S)-norketamine’s *C*_max_ as described above.

### Model redevelopment

The next step was to investigate whether the reported bias in (S)-norketamine prediction could be improved through re-estimation of IIV or structural modifications. Population parameters of the (S)-ketamine model of Fanta et al. were fixed to their reported values and IIV was estimated by forward inclusion based on (S)-ketamine concentrations of CHDR1311 and CHDR1016. The model of Fanta et al. originally had implemented allometric scaling of weight for CL and *V*_d_ and as this covariate relationship is also biologically plausible, it was retained during IIV estimation. IIV was included for CL (dOFV = −80.95) and second inter-compartmental clearance (*Q*_p2_, dOFV = −67.08). However, estimation of variance for *Q*_p2_ greatly increased both the condition number and the RSE% values and was therefore not retained in the model. IIV for *V*_d_ (dOFV = −50.97) was included instead as this was the parameter with the second most significant improvement in model fit. Addition of BOV on these parameters did not significantly improve the model. Estimation of covariance between *V*_d_ and CL was not significant (dOFV = −6.22) but was still included, because it decreased the RSE% of the IIV parameter for *V*_d_ and its shrinkage and did not worsen GOF plots. The GOF plots not only showed a homogenous scatter when comparing observed versus predicted data, but also indicated a possible bias over time for (S)-ketamine. In a separate model analysis, no modifications to the structural model could be made to remove this bias suggesting not the model structure or parameters but rather the data itself is responsible for this trend (data not shown). This could potentially be caused by the rather complex infusion schedule of CHDR1016, which generated these artifacts in the data.

Next, the EBEs of the refined (S)-ketamine model were used as input for the (S)-norketamine model of Fanta et al. Population parameters for the (S)-norketamine model remained fixed while IIV was estimated based on (S)-norketamine data of CHDR1311 and CHDR1016. Stepwise addition of IIV to the model resulted in inclusion of IIV for CL (dOFV = −230.82), distribution volume of the first peripheral compartment (*V*_p1_, dOFV = −182.39), and the transit rate constant (*k*_t_, dOFV = −91.69). The eta distribution of the IIV for CL showed a bias which was centered around −0.5 approximately, indicating a structural error in the model. As the model of Fanta et al. had not originally included IIV on these parameters and the structural model parameters needed refinement, it was concluded that the model structure of (S)-norketamine had to be adapted to correctly fit the data.

In order to improve the model fit and reduce the bias observed in the (S)-norketamine *C*_max_, 1-, 2-, and 3-compartmental models with 0-3 transit compartments were explored. A 2-compartmental model without transit compartments resulted in the lowest OFV value and IIV was included for CL (dOFV = −373.28) and *V*_d_ (dOFV = −108.21). Estimation of covariance between these parameters decreased OFV significantly (dOFV = −18.03). As the model structure differed from the original structure proposed by Fanta et al., weight was not included as covariate from the start of model development, but showed a clear correlation after exploration of the EBEs versus weight. Inclusion of allometric scaling improved the model significantly for *V*_d_ (dOFV = −20.06), but resulted in only a limited improvement after addition for CL (dOFV = −6.01). Still, allometric scaling was included for both parameters, as this was in line with the covariate model structure of the parent compound and supported by biological rationale.

**Fig. 5:**
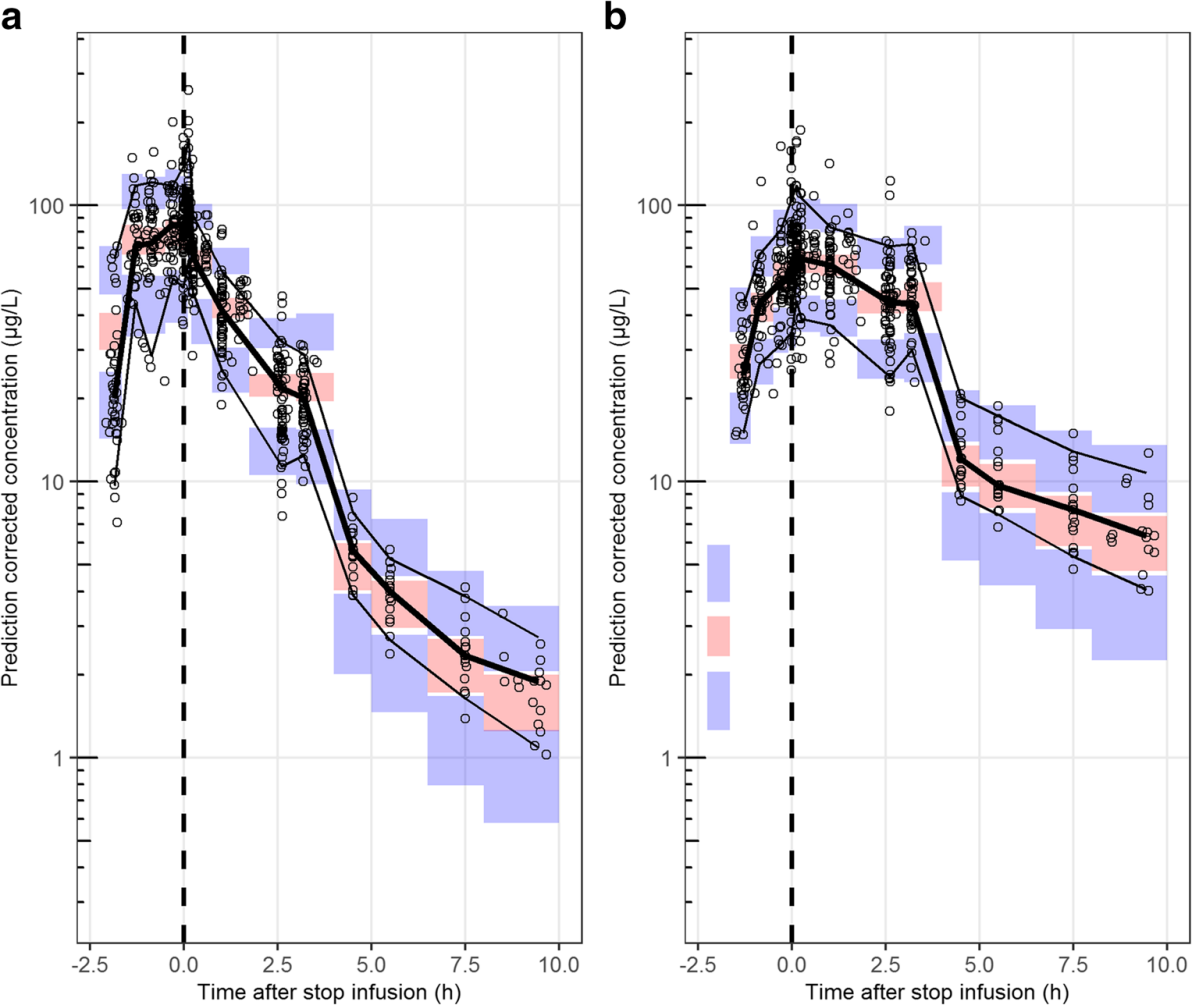
Prediction corrected visual predictive check (pcVPC) of the redeveloped **a** (S)-ketamine and **b** (S)-norketamine model predicting data of CHDR1311 and CHDR1016. The thick and thin black lines represent the median and 80% intervals of observed data. The pink and purple rectangles represent the 95% confidence intervals around the median and 80% prediction intervals of the predicted data. Observed concentrations were corrected for differences in dosing by multiplication with the ratio between the population predicted and the median population predicted value per bin and are shown as open dots

Final model parameters of the sequentially developed models for (S)-ketamine and (S)-norketamine are listed in Table 3. The estimated model parameters were simulated with their corresponding typical value and IIV, which is shown in Fig. 2. The final model structure is illustrated in Fig. 1d. Estimated parameters showed high precision in estimation (RSEs <50%) and the proportional residual error was low. The GOF plots in Figure S2 show a homogenous scatter of population and individual predictions close to the unity line for observed values, for both (S)-ketamine and (S)-norketamine. The majority of the CWRESI values stay within the acceptance interval of −2 to 2. Outliers outside this interval did not significantly alter parameter estimates after removal and were thus retained in the data. The pcVPC in Fig. 5 shows that the model captures the typical PK and corresponding variability for both (S)-ketamine and (S)-norketamine adequately and improved the prediction of (S)-norketamine *C*_max_.

**Table 3:** Pharmacokinetic model parameters for (S)-(nor)ketamine. Population parameters for (S)-ketamine were estimated by Fanta et al. [27]. Population parameters for *V*_d_ and CL were scaled allometrically: θ*(weight/70)^exponent^, where the scaling exponent is 1 for *V*_d_ and 0.75 for CL. Shrinkage (%) of estimated IIV parameters is reported in brackets

	Estimate	RSE%
(S)-ketamine		
Population parameters (*θ*)		
*V*_d_	133	-
CL	95.2	-
*V*_p1_	187	-
*Q*_1_	23.2	-
*V*_p2_	98.8	-
*Q*_2_	157	-
Inter-individual variability (*ω*^2^)		
*V*_d_	0.084 [7.1]	28.4
CL	0.026 [13.2]	21.9
Covariance	0.023	37.8
Residual variability (*σ*^2^)		
Proportional error	0.056	10.3
(S)-norketamine		
Population parameters (*θ*)		
*V*_d_	98.6	3.77
CL	57.7	5.7
*V*_p1_	160	17.6
*Q*_1_	42.8	7.51
Inter-individual variability (*ω*^2^)		
*V*_d_	0.040 [22.9]	29
CL	0.103 [2.8]	22
Covariance	0.044	30.3
Residual variability (*σ*^2^)		
Proportional error	0.020	14.2

The condition numbers of the (S)-ketamine IIV re-estimation and (S)-norketamine model redevelopment were 4.44 and 21.45 respectively

*RSE* relative standard error, *V*_*d*_ distribution volume, *CL* clearance, *V*_*p*_ peripheral compartment, *Q* inter-compartmental clearance

## Discussion

In this study, five previously reported population PK models of (S)-ketamine and (S)-norketamine were compared with each other and externally validated with datasets from two different healthy volunteer studies previously performed at our institution. The model of Fanta et al. provided the best predictions and was further refined with the available data to improve its fit [27]. No structural modifications were required to describe the (S)-ketamine PK, but removal of one peripheral and all transit compartments was necessary to improve the bias in *C*_max_ prediction of (S)-norketamine.

Population PK models of (S)-(nor)ketamine were selected from the literature for external validation based upon predetermined criteria. The exploration of literature models is one of the first steps to perform when simulating a future clinical trial. These results show that when studying (S)-ketamine administration in healthy volunteers, the model of Dahan et al. should be excluded, as their study design led to high parameter variability, which not unexpectedly resulted in a low predictive performance of their model [30]. Nonetheless, our results show that even when population demographics were similar, the derived model structures, parameters, and resulting simulations of the concentrations over time resulted in a wide range, highlighting the need for a detailed comparison of models as was performed in this study. This research further accentuates that purely selecting literature models based on the population on which they were build should not be the only selection criteria.

The outstanding predictive performance of (S)-ketamine concentrations by the model of Fanta et al. coincides with their estimated central *V*_d_ being largest of all five models (133 L). This could have resulted from the use of data after bolus injection for model development, allowing unbiased estimation of the absolute *V*_d_ when measurements are being taken directly after administration. Still, a different 3-compartmental model developed by Geisslinger et al. reported a central *V*_d_ value after bolus injection closer to Sigtermans et al., Noppers et al., and Jonkman et al. (27.9 L, 15.4 L, 17.0 L, and 7.2 L respectively) [18, 21, 26, 31]. The meta-analysis PK model of Kamp et al. also has a central *V*_d_ value of 25 L [25].

A more likely explanation, however, would be the relation between circulatory dynamics right after or during administration and the type of blood sampling, being either arterial or venous. As shown by Henthorn et al. and Kamp et al., (S)-ketamine concentrations measured from arterial samples are systemically higher than venous samples until stop of infusion [25, 28]. CHDR1311, CHDR1016, and Fanta et al. each performed venous sampling, whereas Sigtermans et al., Noppers et al., and Jonkman et al. used arterial blood samples [15, 18, 26, 27, 31, 38]. The PK models of Henthorn et al. and Kamp et al. account for these differences due to measurement type, but as they did not describe (S)-norketamine PK, they did not meet the criteria to be included in this study.

Even though the PK model of Fanta et al. performed best compared to the other models, (S)-norketamine *C*_max_ was still predicted incorrectly. This underlines the necessity of validation and understanding where inter-study differences originate from, as such inaccuracies could greatly affect study outcomes in case the therapeutic window is not reached or exceeded. To provide accurate predictions on (S)-(nor)ketamine PK for future studies on this matter, the (S)-norketamine model of Fanta et al. was modified which resulted in not only increased accuracy but also less complexity than originally described [27]. Compared to their study design, less measurements were taken during the formation and elimination phase of (S)-norketamine in both CHDR1311 and CHDR1016, which not only rationalizes this necessary simplification of the model, but also means that predictions during these phases should be interpreted with care [15, 38]. The estimated (S)-norketamine CL of the redeveloped model is close to the value reported by Fanta et al., yet the estimated *V*_d_ and *Q*_p1_ are much higher and lower respectively. This indicates that the *C*_max_ underprediction by the model of Fanta et al. most likely resulted from the quick redistribution to peripheral compartments.

Lastly, it is worth mentioning that all discussed models, both those selected from literature and the modified model presented here, include assumptions due to parameter identifiability for (S)-norketamine. Metabolite formation and the metabolite volume of distribution cannot be estimated simultaneously and this can only be resolved by assuming that the fraction of (S)-ketamine metabolized to (S)-norketamine is 100% or that *V*_d_ for parent and metabolite are equal. Noppers et al. and Sigtermans et al. even had to include both assumptions [18, 31]. A simple solution to this problem would be to measure the excreted fraction of the drug in urine which would allow for determination of the metabolized and excreted fractions of the compounds [44].

## Conclusion

This study highlighted the vast variability that is present in available literature population PK models for (S)-ketamine, externally validated the simulated concentrations with two clinical datasets, and critically reviewed the presented models. The importance of validation in the cycle of model development was demonstrated and an improved population PK model to predict (S)-(nor)ketamine concentrations after intravenous infusion of (S)-ketamine in healthy volunteers was presented. This model can be used to design future clinical trials on (S)-(nor)ketamine effects on pain and depression, for instance by providing a quantitative rationale for the required dosing regimen to reach a predetermined therapeutic window. To enhance application of (S)-(nor)ketamine population PK models in general, future modeling studies should pay attention to possible differences in PK parameters when given as racemate or pure (S)-enantiomer, include circulatory dynamics to predict both venous and arterial samples correctly, and collect urine data to reduce the number of assumptions necessary for norketamine parameter estimation.

## Supplementary information

Figure S1 Prediction corrected visual predictive check (pcVPC) of (S)-(nor)ketamine model predictions for CHDR1016 data. The model used for predictions of (A.) (S)-ketamine and (B.) (S)-norketamine was copied from Fanta et al. (2015) [27]. The thick and thin black lines represent the median and 80% intervals of observed data. The pink and purple rectangles represent the 95% confidence intervals around the median and 80% prediction intervals of the predicted data. Observed concentrations were corrected for differences in dosing by multiplication with the ratio between the population predicted and the median population predicted value per bin and are shown as open dots. (PNG 75 kb)

Figure S2 Goodness-of-fit plots of the final (A.) (S)-ketamine and (B.) (S)-norketamine model predictions based on data of CHDR1311 and CHDR1016. (1.) Predicted versus observed concentrations, (2.) individual predicted versus observed concentrations, (3.) conditional weighted residuals with interaction (CWRESI) versus predicted concentrations and (4.) versus time after stop of infusion. The red lines are regression lines (1,2) or smooth curves (3,4) (PNG 214 kb)

ESM 1 (TXT 2 kb)

## Data Availability

The data that support the findings of this study are available from the corresponding author upon reasonable request.

## Code availability

Code for simulations with the modified model is available in the supplementary files.
