# Supplementary figures and images for: Predictive performance of parent-metabolite population pharmacokinetic models of (S)-ketamine in healthy volunteers
Source: Eur J Clin Pharmacol. 2021 Feb 11;77(8):1181–92. doi: 10.1007/s00228-021-03104-1 (PMC8275530; doi:10.1007/s00228-021-03104-1)

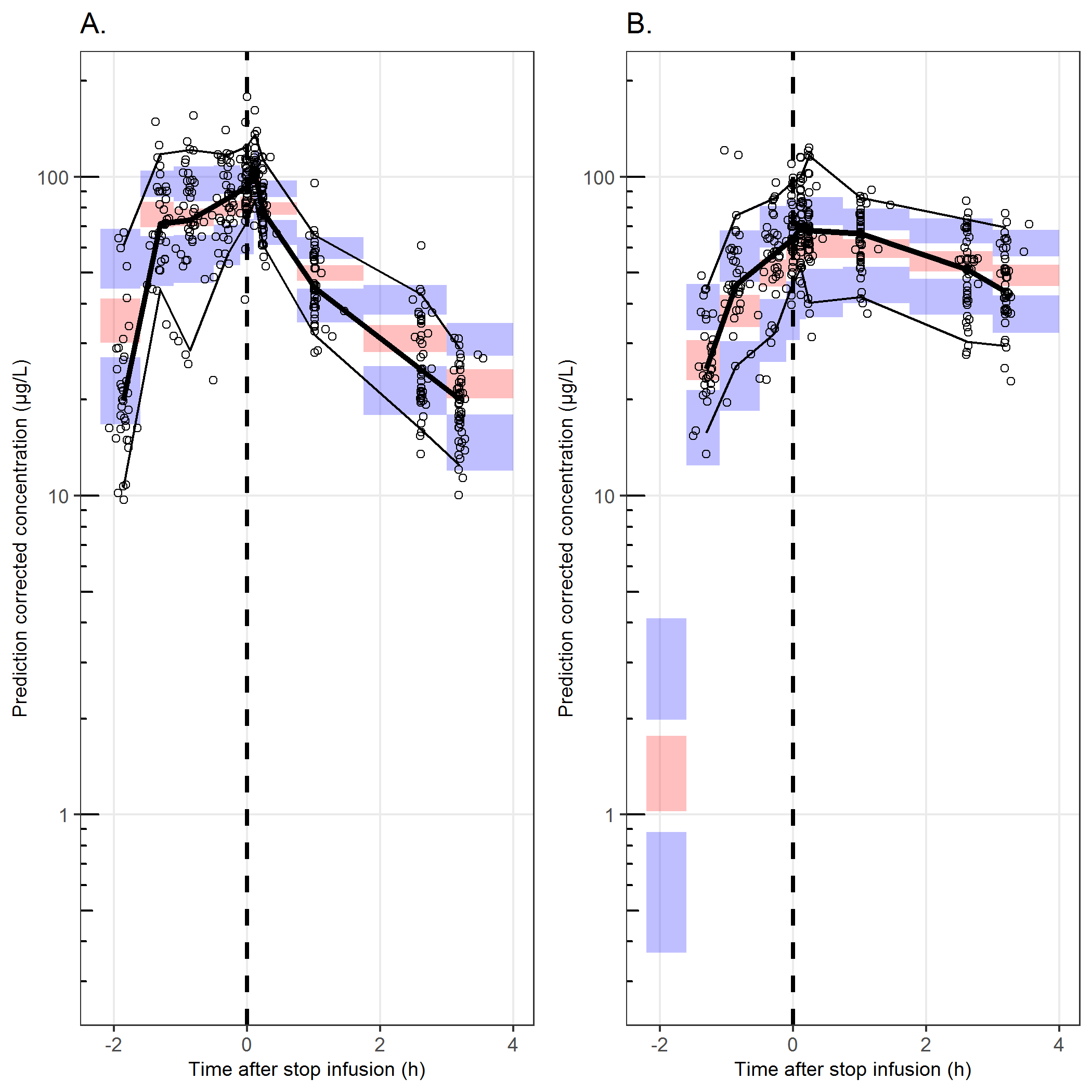

Supplement: Supplementary file 1 — Prediction corrected visual predictive check (pcVPC) of (S)-(nor)ketamine model predictions for CHDR1016 data. The model used for predictions of (A.) (S)-ketamine and (B.) (S)-norketamine was copied from Fanta et al. (2015) [27]. The thick and thin black lines represent the median and 80% intervals of observed data. The pink and purple rectangles represent the 95% confidence intervals around the median and 80% prediction intervals of the predicted data. Observed concentrations were corrected for differences in dosing by multiplication with the ratio between the population predicted and the median population predicted value per bin and are shown as open dots. (PNG 75 kb) [file 228_2021_3104_MOESM1_ESM.png]

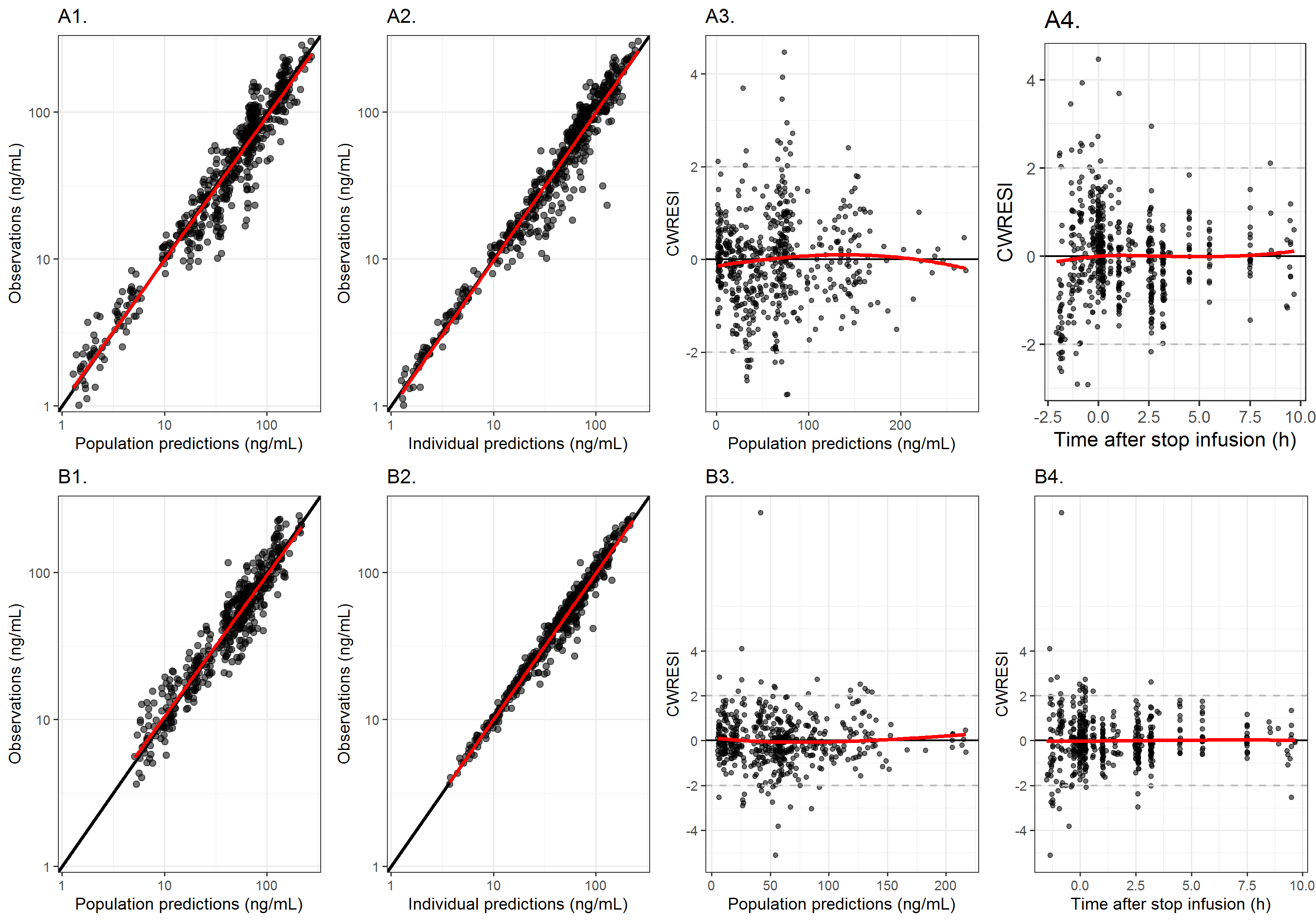

Supplement: Supplementary file 2 — Goodness-of-fit plots of the final (A.) (S)-ketamine and (B.) (S)-norketamine model predictions based on data of CHDR1311 and CHDR1016. (1.) Predicted versus observed concentrations, (2.) individual predicted versus observed concentrations, (3.) conditional weighted residuals with interaction (CWRESI) versus predicted concentrations and (4.) versus time after stop of infusion. The red lines are regression lines (1,2) or smooth curves (3,4) (PNG 214 kb) [file 228_2021_3104_MOESM2_ESM.png]
